# Clinical Characteristics and Current Status of Treatment for Recurrent Bladder Cancer after Surgeries on Upper Tract Urothelial Carcinoma

**DOI:** 10.3390/diagnostics13051004

**Published:** 2023-03-06

**Authors:** Xinfeng Hu, Yufan Xue, Guodong Zhu

**Affiliations:** Department of Urology, The First Affiliated Hospital of Xi’an Jiaotong University, Xi’an 710061, China; wangwxpp@gmail.com (X.H.); yukixyf00@gmail.com (Y.X.)

**Keywords:** upper tract urothelial carcinoma, intravesical recurrence, non-muscle invasive bladder cancer, risk factor, treatment

## Abstract

Upper tract urothelial carcinoma (UTUC) is a relatively rare, but highly malignant, disease with an estimated annual incidence of 2 cases per 100,000 people. The main surgical treatment modalities for UTUC are radical nephroureterectomy (RNU) with bladder cuff resection. After surgery, intravesical recurrence (IVR) can occur in up to 47% of patients, and 75% of them present with non-muscle invasive bladder cancer (NMIBC). However, there are few studies focused on the diagnosis and treatment of postoperatively recurrent bladder cancer for patients with previous UTUC history (UTUC-BC), and many of the influencing factors are still controversial. In this article, we performed a narrative review of the recent literature, mainly summarizing the factors influencing postoperative IVR in patients with UTUC and discussing the subsequent prevention, monitoring, and treatment tools for it.

## 1. Clonogenic Correlation and Tumor Implantation Theory

**Figure 1:**
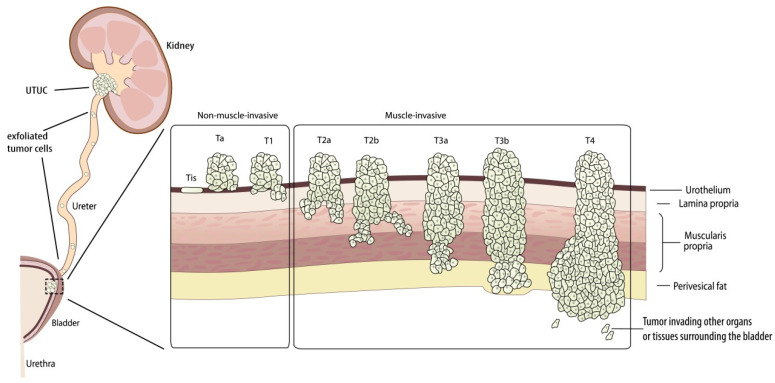
The colonial origin and intracavitary seeding theory of postoperatively recurrent bladder cancer for patients with upper tract urothelial carcinoma history (UTUC-BC).

It remains controversial whether upper tract urothelial carcinoma (UTUC), and subsequently, urinary bladder cancer (BC) are of clonally related or separate origins. Several studies in recent years have supported a clonal origin with intratumoral implantation. According to Fadl et al., the presence of related clones with high karyotypic similarity in anatomically distinct tumors from the same bladder suggests that multifocal urothelial tumors have a monoclonal origin and arise by intraluminal inoculation of living cancer cells shed from the original tumor [1]. There is a tendency for UTUC to recur around the cystostomy tube wall or within the bladder neck where the urethral duct is damaged, which further supports the hypothesis that cancer cells floating in the bladder may primarily adhere to the injured urethra and recur through intraluminal inoculation [2]. Habuchi and colleagues [3] found that upper urinary tract and bladder tumors from the same patient consistently exhibited the same distinct p53 mutation. Doeveren et al. systematically reviewed the available relevant literature on the possible clonal relationship between UTUC and BC, and they suggested that 94% of primary UTUC and intravesical recurrences (IVR) are clonally related [4]. To further investigate the clonal relationship between the two entities, Audenet F et al. investigated the genes from UTUC and the specific recurrent BC tissue specimens from 29 patients by using somatic mutation data to study their clonal correlation. It was found that all the UTUC and BC pairs were considered to have similar clonal origins (*p* < 0.005) [5]. Additionally, Doeveren et al. [6] performed a targeted DNA sequencing technique on a panel including 41 genes, and the results showed that 73.3% of patients with paired UTUC and BC exhibited the same clonal relationship. Aside from that, the sample they took were from patients who had been diagnosed with primary urothelial carcinoma of the upper urinary tract, and subsequently diagnosed with urothelial carcinoma of the bladder, and this approach more accurately reflects the natural course of patients with UTUC after surgical treatment. This result supported the hypothesis that recurrent BC was primarily a clonally derived recurrence after the primary surgical resection of UTUC, rather than a separate entity. During the follow-up, three patients in their cohort developed multiple recurrent BCs, which were all associated with primary UTUC, and thus, provide further support for the possible mechanism of the tumor cells seeding theory (see Figure 1).

Based on the clonal origin, there was 80% concordance between the tumor grading of primary UTUC and recurrent BC in up to 90% of cases [7]. However, in contrast to grading, the pathological stage of UTUC was poorly correlated with that of recurrent BC (UTUC-BC). In the study by Raman and colleagues, 92% of bladder recurrences in patients with superficial or invasive UTUC were of superficial bladder cancer [7], and almost all the UTUC-BCs tended to be superficial tumors independent of the stage of the primary UTUC tumors [7,8].

## 2. Comparison of the Characteristics of Recurrent and Primary BC

Currently, the disease management of patients with IVR after radical nephroureterectomy (RNU) for UTUC (UTUC-BC) is based on the primary BC guidelines. For non-muscle invasive bladder cancer (NMIBC), the transurethral resection of bladder tumor (TUR-BT) remains the initial management option. However, according to a large population-based survey by Wu et al. [9], the baseline characteristics of the two patient’s cohorts with recurrent BC after UTUC (UTUC-BC) and patients with primary BC were so different that the treatment guidelines for patients with primary BC were not fully applicable to the patients with UTUC-BC. For the UTUC-BC patients’ cohort, the majority of the patients were white (88.0%), male (58.7%), with a lower proportion of females (41.3%) and those with an earlier TNM stage. The median age of the patients with IVR was 72.07 years old, and the median BC tumor size was 24.36 mm. Compared to UTUC-BC patients, primary BC patients were more likely to be male (76.7%), with a larger median tumor size (34.84 mm) and earlier TNM stages (*p* < 0.001). The primary sites of tumor location were significantly different between the UTUC-BC and primary BC patients (*p* < 0.001), with the most common sites for UTUC-BC being the lateral wall and bladder neck, mostly presenting as NMIBC [10], while the primary BC patients were more likely to have tumors in the trigone of the bladder. For primary UTUC lesions in patients with UTUC-BC, the highest proportion of patients had renal pelvic carcinoma (74.7%), grade III/IV (67.6%), and stage N0 (91.0%).

The BC seems to be more difficult to treat than UTUC-BC does in terms of size and staging, but Miyake M et al. found that UTUC-BC had a worse prognosis with bacillus calmette-guérin (BCG) instillation in the bladder compared to that of primary BC, suggesting that these recurrent tumors inherently respond poorly to BCG [11]. Meanwhile, Shigeta K et al. also observed that the fibroblast growth factor receptor 3 (FGFR3) level was significantly lower in primary MIBC patients than it was in UTUC-BC patients (including NMIBC and MIBC, *p* < 0.01). In contrast, MIBC specimens (including IVR and primary BC) showed a higher expression of P53 levels than those of IVR of NMIBC specimens (*p* = 0.03 and 0.04, respectively) [12]. Increased expression of FGFR3 and P53 is frequently associated with tumor cell generation and progression, thus UTUC-BC might have characteristics such as being more aggressive in terms of growth and invasion. Interestingly, one study conducted by Wu et al., who investigated the prognosis of patients with BC, found that the cancer-specific survival (CSS) of UTUC-BC patients was not significantly different from the CSS of primary BC patients [9]. However, the CSS of the former group (11.4%) was significantly higher than that of the latter group (0.7%). Due to the impact of UTUC, the overall prognosis of UTUC-BC patients was worse than that of primary BC patients. The median survival times for UTUC-BC patients and primary BC patients were 54 and 97 months, respectively (*p* < 0.001). For the type of NMIBC, the median survival rates were 67 and 112 months for UTUC-BC and primary BC patients, respectively (*p* < 0.001) [9]. More importantly, the results demonstrated that neither radical cystectomy nor TUR-BT could provide a significant survival benefit for patients with UTUC-BC compared to that of the patients with primary BC who received the same surgical treatment. The study by Yates et al. indicated significant differences in the genetic and epigenetic background between the patients with UTUC-BC and primary BC [13], and these differences might be one of the factors that could result in the different treatment effects for the two patient cohorts with the same treatment strategy. Meanwhile, Makito et al. identified that patients with UTUC were more likely to develop IVR NMIBC after receiving intravesical BCG instillation compared with the likelihood of patients with primary NMIBC after matching UTUC-BC and primary NMIBC patients according to their propensity scores [11]. This was consistent with other existing studies and suggested that while BCG was currently one of the most effective intravesical agents for preventing recurrence of NMIBC, its role in disease progression still remained controversial.

## 3. Risk Factors Affecting Recurrent BC

The main surgical treatment modalities for UTUC are radical nephroureterectomy (RNU) with bladder cuff resection [14]. Patients with UTUC have a higher risk of tumor recurrence after receiving surgery, such as recurrence in the bladder and local or distant metastasis, which can be as high as 47%, 18%, and 17%, respectively [15,16]. Of the patients who experienced intravesical recurrence (IVR) during follow-up, 75% presented with non-muscle invasive bladder cancer (NMIBC), which was confined in the mucosa (Ta, CIS) or submucosa (T1) of the bladder wall [17]. Therefore, exploring the risk factors affecting the postoperative IVR for patients with UTUC is essential for subsequent monitoring and treatment.

However, not all patients with UTUC are suitable for the risk factor assessment. For example, some studies have shown that when UTUC tumors are first diagnosed, 60% of them are aggressive, and nearly 25% of them are regionally metastatic [18]. Aggressive and late-staged tumors might indicate difficulties during the treatment with a poor prognosis, and they also represent a higher possibility for metastasis. For UTUC that is aggressive, the 5 year specific survival rate is <50% for patients with pT2/pT3 staging and <10% for those with pT4 staging [19]. Therefore, when one is treating patients with aggressive or advanced UTUC, the goal of the treatment for physicians is to improve the life quality and prolong survival time for the patients, while monitoring or treating their metastases, but the management for the possible IVR is not a primary consideration. In the following part regarding risk factors that could affect the incidence of recurrent BC for UTUC patients, we only focus on patients with staging lower than pT2.

As shown in Table 1, we can largely classify the factors that may influence the incidence of postoperative IVR for patients with UTUC after receiving RNU into four categories.

### 3.1. Patient-Specific Factors

#### 3.1.1. Damaged eGFR

Reduced eGFR may bring about electrolyte disturbances and imbalances of the internal environment within the patient. Kuroda K et al. retrospectively studied 187 UTUC patients with RNU and they found that a preoperative eGFR < 60 mL/min/1.73 m^2^ (HR = 2362, 95% CI = 1067–5592) is an independent factor for higher IVR in all UTUC patients [20]. In addition, they believed that chronic kidney disease (CKD) or end-stage renal disease might be associated with the progression or invasiveness of UTUC [20]. Similarly, Momota M et al. analyzed the clinical data of 1066 patients with UTUC, and the Cox analysis showed that there was a significant correlation between eGFR < 45 mL/min/1.73 m^2^ and IVR before RNU. These results might be due to the low eGFR, and some studies have found that the eGFR in UTUC patients could be decreased by 18% after RNU [21]. Some studies demonstrated that CKD could lead to chronic inflammation, oxidative stress, metabolic disorders, and uremia-related immunodeficiency, which promote immune escape and the growth and metastasis of tumors [22,23].

#### 3.1.2. Venerable Age

Xylinas E et al. analyzed data from 1261 UTUC patients with RNU, and their multivariate Cox regression analysis showed that advanced age is associated with the postoperative IVR (*p* = 0.03) [15]. Similarly, a study by Chromecki TF et al., who analyzed data from 1169 UTUC patients, they found that an age > 70 years old is an independent predictor for UTUC recurrence (*p* = 0.018), and they also found a 40.2% probability of recurrence in UTUC patients older than 80 years old in the third year after RNU [24]. However, when these data were categorized by the physical performance status, the multivariate analysis found that age is not associated with disease recurrence (HR = 1.38, *p* = 0.101) [24]. This observation suggested that UTUC recurrence might be more influenced by the physical health of the patient, but less related to the actual physical age. The study by Shariat SF et al. included 1453 patients with UTUC, and their multivariate analysis indicated that advanced age is not associated with recurrence of UTUC [25]. However, they found that an older age is associated with history of previous ureteroscopy, a history of BC, an infiltrative tumor structure, and a poorer physical performance status in the investigated data. These above factors might contribute to the high recurrence rate of UTUC. Therefore, in actual clinical practice, the possibility of recurrence in patients of old age still needs to be considered in a focused manner.

#### 3.1.3. Gender Difference

UTUC is usually prevalent in men; however, Chien TM et al. analyzed data from 368 Chinese UTUC patients and they found a higher incidence of UTUC in women with advanced CKD. Multifactorial analysis showed that advanced CKD is an independent predictor for recurrent-free survival (RFS) in women with UTUC [26]. In fact, in China, some women use traditional herbal medicines containing aristolochic acid during pregnancy or when they suffer from some diseases. It has been suggested that exposure to aristolochic acid is an important reason for the high incidence of UTUC in Chinese women [27], so it is also likely that the occurrence of IVR in some women with UTUC history is due to excessive aristolochic acid consumption. In one study by Xylinas E et al. and Ploussard G et al., they found that being male is associated with the occurrence of IVR for UTUC patients (*p* = 0.04 and 0.003, respectively) [15,28]. Seisen T et al. conducted a meta-analysis including 18 studies with a total of 8275 UTUC patients, and after multifactorial analysis, they found that being male is a significant predictor of the postoperative IVR (HR = 1.37, *p* < 0.001) [29]. Therefore, some researchers argued that adequate treatment and strict monitoring were necessary to reduce the tumor recurrence when one is dealing with male UTUC patients.

#### 3.1.4. Smoking

Smoking status or cumulative exposure has previously been shown to be associated with bladder recurrence after RNU [15]. Xylinas E et al. performed a clinical trial including 519 UTUC patients after RNU. They classified the patients by current smoking status, cigarettes consumption per day, smoking duration, and time until they had quit. Using multivariate analysis, they found that current smoking duration (≥20 years) and heavy long-term smoking were associated with a higher risk of IVR (both *p* ≤ 0.04). In addition to this, patients who quit smoking ten years before receiving RNU had a lower risk of IVR than those who did not quit did [30]. Similarly, Crivelli J.J. et al. performed a meta-analysis of three studies on smoking and showed that smoking is associated with IVR in two of the studies [31]. In one study by Ehdaie B et al., who analyzed the disease characteristics of 288 UTUC patients, they found that smoking status is not associated with the risk of UTUC recurrence or death (*p* = 0.60). However, the risk of death is significantly higher in smokers than it is in non-smokers (HR = 3.64, 95% CI = 1.59–8.34) [32]. Therefore, persuasive smoking cessation should be of great concern to surgeons, both in terms of the recurrence and prognosis for patients with UTUC.

#### 3.1.5. Diabetes Mellitus with Poor Glycemic Control

Poor glycemic control is most often seen in diabetic patients. Recent studies suggest that not all UTUC patients with diabetes will have a higher risk for IVR. Data from a study including 538 UTUC patients showed that diabetic patients with poor glycemic control (HbA1c ≥ 7.0%) exhibited a shorter RFS for recurrent bladder cancer compared with those of diabetic and non-diabetic patients with good glycemic control (both *p* < 0.001). In addition, in a multivariate analysis, poor glycemic control in diabetes independently predicted IVR (HR = 2.10, *p* < 0.018) [33]. Similarly, a meta-analysis including 10 studies demonstrated that diabetes could increase the risk for IVR in patients with UTUC (HR = 1.26, 95% CI = 1.11–1.43, *p* = 0.0004) [34]. It has been shown that hyperglycemia not only provided more nutrients to tumor cells, but also decreased the immunity, so that poor glycemic management in UTUC patients could contribute to tumorigenesis, apoptotic resistance, and resistance to chemotherapy [35].

#### 3.1.6. Monocyte-to-Lymphocyte Ratio (MLR)

The increasing numbers of studies have shown that inflammation might be associated with the survival and progression for malignant tumors [36]. Therefore, it is feasible to examine cancer patients for inflammatory aspects to determine their specific prognosis. The monocyte-to-lymphocyte ratio (MLR) has been shown to correlate with the outcome of patients with UTUC [37]. A multivariate analysis by Liu J et al., who analyzed data from 441 UTUC patients after receiving RNU, found that the preoperative MLR > 0.22 is significantly associated with IVR (HR = 4.085, *p* < 0.001). These results might suggest that MLR is an independent predictor for postoperative IVR in patients with UTUC [36].

#### 3.1.7. Neutrophil-to-Lymphocyte Ratio (NLR)

In addition to monocytes, the number of neutrophils is also used as an indicator for inflammation in the organism, and an increased neutrophil count often represents the development of inflammation [38]. De Larco et al. showed that neutrophils in the tumor microenvironment could play a key role in angiogenesis and cancer progression [39]. In addition to this, lymphocyte reduction may cause further immune escape for tumor cells. Therefore, an increased neutrophil-to-lymphocyte ratio (NLR) may suggest a higher chance of tumor invasion and metastasis and may cause IVR for UTUC patients after surgery. Vartolomei et al. analyzed nine studies including 4385 UTUC patients, and out of the six NLR-related studies, five demonstrated that elevated NLR is an independent predictor for tumor recurrence after patients receiving RNU (HR = 1.60, 95% CI = 1.16–2.19, *p* = 0.004) [40]. Similarly, a study including 2477 UTUC patients demonstrated that patients with an NLR > 2.7 had a worse RFS than the patients with normal NLR did using an univariate analysis (*p* < 0.003), but no statistically significant difference was found in a multivariate analysis (*p* = 0.59) [41]. Consequently, not all the studies demonstrated that an elevated NLR could independently predict postoperative recurrence in patients with UTUC, and their findings require further analysis. In actual clinical practice, when they are treating UTUC patients with an elevated NLR, physicians should proactively consider the possible elevated risk for tumor recurrence for their patients after receiving RNU.

### 3.2. Tumor-Specific Factors

#### 3.2.1. Multifocality of Upper Urinary Tract Tumors

Tumor multifocality refers to the presence of multiple tumor lesions in the unilateral urinary tract, which is often considered to have a worse prognosis than a single tumor does. In a retrospective review of 342 patients with UTUC, Milojevic B et al. found that tumor multimodality is associated with RFS (HR = 2.86, 95% CI = 2.06–3.99, *p* < 0.001) [42]. In terms of IVR, Milojevic B’s study showed the same results. Their multivariate analysis showed that tumor multiplicity is the only significant factor for predicting IVR (HR = 1.40, *p* = 0.037) [43]. In clinical practice, tumor multiplicity of UTUC can be divided into multiple renal pelvic tumors, multiple ureteral tumors, and synchronous renal pelvic and ureteral tumors. Chen CS et al. retrospectively analyzed the data from 685 patients with UTUC diagnosed with multiple tumors, and they found that the synchronous renal pelvic and ureteral tumors group had a higher probability of IVR than the multiple renal pelvic tumor group did (*p* = 0.018) [44]. This result suggested that UTUC patients with tumors in both the renal pelvis and ureter might require more stringent treatment and monitoring. The susceptibility to recurrence might be explained by the fact that multiple tumors tend to possess a more aggressive oncologic behavior and are more likely to be missed or delayed in the process of diagnosis and treatment [45].

#### 3.2.2. Size of UTUC

Tumor size is generally the main factor to describe the nature of tumors, and it has been previously demonstrated that a larger UTUC tumor might have a higher risk for IVR [46]. In a multivariate analysis, Shibing Y et al. retrospectively analyzed data from 795 patients with UTUC, and showed that a tumor > 3.0 cm is an independent predictor for RFS (HR = 2193, *p* < 0.001) [47]. The same conclusion was reached in the study by Espiritu PN et al. (HR = 1.97, *p* = 0.011), and they also found 5 year RFS rates were 46.9% and 25.8% for patients with tumor sizes < 3 cm and ≥3 cm, respectively [48]. This result might be explained by the fact that oversized UTUC tumors were not only more invasive, but also compressed or even obstructed the upper urethra resulting in high pressure in the upper urethra, where tumor cells might be more likely to be shed, invade, and implanted into the bladder. In contrast, a multifactorial analysis including 687 UTUC patients found that the effect of tumor size on IVR is not significant. However, the tumor size > 3 cm is significantly associated with IVR in a univariate analysis (*p* = 0.011) [49]. Therefore, UTUC patients with a tumor size > 3 cm still need to be given more attention in clinical practice.

#### 3.2.3. Distal Ureteral Position

Tumors in UTUC are usually classified as tumors located in the ureter, renal pelvis, and multiple site tumors. It has been shown that tumors in the lower/middle ureter have a higher rate of local recurrence, but tumors in the renal pelvis have a higher prevalence of metastasis in distant organs such as the lungs [16]. Therefore, it is likely that UTUC tumors in different locations have different effects on IVR. Xylinas E et al. analyzed 1839 patients with UTUC, and they found that a tumor located in the ureter is significantly associated with IVR (*p* = 0.03) [15]. In addition to this, the same conclusion was reached by other two multivariate meta-analyses, the authors of which concluded that ureteral location is a significant predictor for IVR (both *p* < 0.001) [29,50]. The reason for this result might be that ureteral tumors could cause ureteral obstruction even at earlier stages and grades [50], and that the higher pressure of the ureter and its closer proximity to the bladder result in a higher probability of seeding tumor cells.

#### 3.2.4. Lymph Node Involvement

Regional lymph nodes are the most common site of metastasis for UTUC patients [51], and tumor grading and lymph node involvement often represent increased malignancy and a higher probability of metastasis [52]. Novara et al. demonstrated that lymph node involvement is an independent predictor for CSS, and they believed that UTUC patients with lymph node involvement had a three-fold increased risk of death compared to that of the patients with lymph node-negative disease [53]. Therefore, the prevention of IVR in UTUC patients is not necessarily a primary consideration for patients with regional lymph node positive disease, and physicians should pay close attention to the complete removal of regional lymph nodes and tumor-containing tissues during the surgery and the possible local recurrence or distant metastasis when they follow up with the patients. For UTUC patients, lymph node involvement is likely to indicate that tumor cells are more likely to seed into the bladder. In a multivariable Cox regression analysis, Xylinas E found that lymph node involvement is independently associated with the occurrence of IVR for patients with UTUC (HR = 1.69, 95% CI = 1.19–2.40, *p* = 0.003) [15]. Verhoest G et al. reached a similar conclusion that the proactive and appropriate lymph node dissection could improve the specific survival for UTUC patients [54]. However, lymph node dissection may also have adverse effects on patients, leading to prolonged operative time and increased postoperative complications. Since the main site of lymph node metastasis depends on the location of the primary tumor [55], there is no exact standard for reginal lymph node dissections in different locations of UTUC, and therefore, individualized consideration for patients with lymph node involvement is needed.

#### 3.2.5. Invasive pT Staging

For UTUC patients, a large part of the occurrence of IVR may be due to excessive ureteral pressure caused by oversized tumors, which can seed cancer cells through the ureter into the bladder. In a multivariate analysis, Seisen T et al. retrospectively reviewed the data from 5041 patients with UTUC and found that the advanced stage (pT2, pT3, or pT4) is a significant predictor for IVR (HR 1.38, 95% CI 1.20–1.60; *p* < 0.001) [29]. Additionally, Verhoest G et al. and Li Y et al. also found that factors such as locally aggressive budding tumors (i.e., aggressive pT staging) could increase the likelihood of cancer cells seeding into the bladder, and they were associated with the development of recurrent BC after the primary UTUC was completely resected [54,56]. Therefore, UTUC at late stages not only have a higher malignancy, but also may cause excessive ureteral pressure or even obstruction, which may promote tumor cells implantation and lead to IVR.

#### 3.2.6. Papillary Structure of Tumors

Approximately two-thirds of UTUC patients have tumors with a papillary growth pattern and one-third have a sessile growth pattern. Different tumor structures are likely to possess different oncological behaviors. The study by Remzi M et al. included 1363 patients with UTUC after RNU, and they found the sessile tumor architecture is an independent factor for cancer recurrence (HR = 1.5, *p* = 0.002) and cancer-specific mortality (HR = 1.6, *p* = 0.001) [57]. This was similar to the findings by Fritsche HM et al., who suggested that the sessile structure of the tumor is a predictor for IVR [58]. Indeed, sessile carcinomas are more likely to suggest muscle-infiltrating disease, more aggressive behavior [57], a worse staging, and vicious oncological behavior. However, in recent years, a different opinion was presented by Ishioka J et al., who selected 754 UTUC patients for a multifactorial analysis, and they concluded that the papillary structure of the tumors is a predictor for IVR (HR = 1.676, 95% CI = 1087–2585, *p* = 0.019) [59]. Therefore, a deeper investigation is needed regarding the effect of tumor structure for the occurrence of IVR.

#### 3.2.7. Extensive Tumor Necrosis

The meta-analysis by Seisen T, including 303 UTUC patients, showed that tumor necrosis is a significant predictor for IVR (HR = 2.17, 95% CI = 1.11–4.26, *p* = 0.02) [29]. Several other studies with large samples showed that extensive tumor necrosis is independently associated with disease recurrence and survival [60,61]. In the clinical setting, tumor necrosis has been shown to be associated with a poor outcome in many cancers [62], and it might indicate high malignancy and overgrowth of the tumor. Therefore, one possible explanation for the above results might be that the partially detached necrotic tumors are more likely to metastasize and implant due to the flow of urine in the renal pelvis and ureter.

#### 3.2.8. Concomitant Carcinoma In Situ (CIS)

CIS is a cytologic lesion which occurs in the uroepithelium and basement membrane and has the potential to infiltrate and invade into deep layer tissues. Concomitant CIS is defined as the presence of CIS associated with another pathological stage. In a multivariate analysis, Wheat JC et al. grouped 1387 patients with UTUC according to CIS, non-CIS, and concomitant CIS, and they found that concomitant CIS is a predictor for the development of IVR in patients with UTUC (HR = 1.25, *p* = 0.04) [63]. Furthermore, in a retrospective study, the prevalence of combined CIS in patients with UTUC was 27–36% [64], and CIS has been long associated with aggressive diseases. It was shown that patients with CIS present at the time of the initial diagnosis were more likely to develop an aggressive disease if they were not treated promptly [63]. The same conclusion was reached by Otto W et al., who performed a multivariate Cox regression analysis using data from 772 UTUC patients, and they found that concomitant CIS is an independent predictor for RFS (HR = 1.9, *p* = 0.007) and CSS (HR = 1.7, *p* = 0.048) [83]. The above studies illustrated that concomitant CIS in UTUC patients not only cause tumor invasion and recurrence, but more importantly, if it is not promptly treated, it could also seriously affect the life expectancy of the patients.

### 3.3. Treatment-Specific Factors

#### 3.3.1. Incomplete Excision

The inadequate surgical treatment of UTUC is a clear predictor for ipsilateral ureteral stump or bladder tumor recurrence, such as incomplete resection, which increases the risk for IVR [46]. Meanwhile, the study by Zou et al. and Chung JH et al. confirmed that incomplete resections in patients were significantly associated with their IVR [65,66]. Seisen T et al. performed a meta-analysis using 18 retrospective studies including 8275 patients with UTUC, and the results demonstrated that an incomplete resection is a significant predictor for IVR (HR = 1.90, *p* = 0.004) [29]. Indeed, due to surgical disruption, residual tumors were unstable; therefore, according to the implantation theory, their shed tumor cells might be more likely to implant through the ureter into the bladder, eventually causing IVR.

#### 3.3.2. Immature Laparoscopic Technique

Furthermore, different surgical choices contribute to variable recurrence rates. According to present studies, there was no evidence to confirm that the prognosis of patients treated with laparoscopic RNU (LRNU) was worse than that of those treated with open RNU (ORNU) [67]. A meta-analysis including 10,730 patients with UTUC obtained the same conclusion; however, after multivariate analysis, Piszczek R et al. found that there was no significant difference between LRNU and ORNU in terms of IVR (HR = 1.08, 95% CI = 0.85–1.39, *p* = 0.52) [68]. Nevertheless, there were some arguments against this, a meta-analysis conducted by Seisen T et al. showed that patients treated with LRNU had a significantly increased risk for IVR compared with that of those receiving ORNU (HR = 1.62, 95% CI = 1.18–2.22, *p* = 0.003) [29]. Therefore, the impact of the surgical approach of RNU on IVR requires further study. For the existing medical technology, both LRNU and ORNU are relatively mature [69], and more often, the surgical approach is determined by the patient’s tumor location and shape and the proficiency of the surgeons. Therefore, postoperative tumor metastasis or recurrence is more likely to be determined by the surgical technique. Shigeta K et al. selected 136 UTUC patients for comparison; half of them were treated with pure laparoscopic radical nephroureterectomy (*p*-LRNU) and the other half was treated with conventional LRNU. The clinical data demonstrated that the 3 year IVR-free survival rate in the *p*-LRNU group was 41.8%, which was significantly lower than those in the LRNU group (66.6%, *p* = 0.004). Multifactorial analysis showed that a history of *p*-LRNU is an independent risk factor for subsequent IVR. Thus, unskilled and imperfect laparoscopic technique usually leads to unstable postoperative prognostic outcomes.

#### 3.3.3. Surgery Time

In recent years, it has been found that not only the surgical approach affects the patient’s IVR, but also, the duration of the surgical procedure might be associated with IVR. Shigeta K et al. found that with the longer duration of pneumoperitoneum created by an infusion of pressurized CO_2_ gas at a pressure of 10–12 mmHg during LRNU, the risk of IVR in UTUC patients was higher [70]. Similarly, the Fisher’s exact test analysis by Yanagi M et al. demonstrated that the prolonged duration of pneumoperitoneum at ≥210 min with 8 mmHg CO_2_ gas pressure injected to create pneumoperitoneum during retroperitoneum LRNU was highly correlated with the risk for IVR (*p* = 0.0358). These results might be due to the extrusion of dislodged tumor cells by the high-pressure gas, resulting in the tumor cells implantation into the bladder cavity [71]. Therefore, excessive surgery time might be another risk factor for IVR.

#### 3.3.4. Early Ureteral Ligation

Yamashita S et al. found that the IVR-free survival rates at years one and two for patients with renal pelvic cancer were 89% and 86% in the early ureteral ligation group and 74% and 64% in the control group (*p* = 0.025), respectively, so early ureteral ligation is an independent predictor for IVR in patients with UTUC located in the renal pelvis [72]. Chen MK et al. selected 85 eligible cases, and they found that early ureteral ligation is an independent risk factor for subsequent bladder recurrence after UTUC (HR = 2.35, 95% CI = 1.53–3.48, *p* = 0.041). The IVR rate in patients with early ureteral ligation was significantly lower compared to that of the patients in the standard group (14.3% vs. 34.9%, *p* = 0.026) [73]. During RNU, some surgical movements are very likely to touch or squeeze the tumor tissues, some of the detached tumor cells may then migrate down through the ureter into the bladder. So early ureteral ligation during the operation not only facilitates the operation, but also reduces the probability of IVR.

#### 3.3.5. Ureteroscopy

In addition, cancer cells could easily migrate to other locations within the urinary tract during ureteroscopy (URS). A multifactorial analysis conducted by Sung HH et al. showed that IVR rates were significantly higher in the preoperative ureteroscopic biopsy (URS-Bx) group (HR = 1558, 95% CI = 1204–2016, *p* = 0.001) and did not differ regardless of whether or not manipulations such as tumor biopsy and balloon dilation were performed (*p* = 0.658) [74]. In the same way, Li YR et al. showed that preoperative ureter manipulation is an independent factor for IVR (*p* = 0.005) [56]. Addtionally, Yoo S et al. found that URS us also an important risk factor for IVR in patients with UTUC [75], including the pre-RNU URS (URS with no tissue biopsy) and diagnostic URS (URS with tissue biopsy). Another recent study conducted by Loizzo et al. reached the same conclusion, and they found that the diagnostic accuracy of URS-Bx was only 72.4% for low-grade UTUC and 36% for in-staging accuracy in all grades for UTUC patients [76]. The above four studies suggest that URS should be minimized or avoided if the diagnosis could be confirmed preoperatively by other examination methods; additionally, diagnostic URS should be used to make decisions based on different risk stratification and is more inappropriate for determining tumor staging for patients with UTUC. Nowadays, there are two types of ureteroscopes, including rigid and flexible ones, and the different ureteroscopes might also lead to different risks for IVR. A multivariate Cox regression analysis including 491 UTUC patients by Ha JS et al. showed that the flexible URS group was significantly more associated with an elevated IVR than the non-URS group was (HR = 1807, *p* = 0.0416). In contrast, the difference for IVR between the non-URS and rigid URS groups was not statistically significant (HR = 1301, *p* = 0.3388) [77]. The study suggested that rigid URS might be safer, which could be explained by the fact that flexible URS required a more pressurized irrigation fluid to ensure the field of viewing during examination, thus exacerbating the shedding of tumor cells.

### 3.4. Molecular-Specific Factors

#### 3.4.1. E-Calmodulin

Calmodulins are a group of transmembrane proteins that are reliant on calcium (Ca^2+^) ions for their action and are essential for maintaining cell-to-cell contact and modulating the cytoskeletal complexes. In this group, E-calmodulin plays an important role in cell adhesion, whose loss of expression is a signature of epithelial–mesenchymal transition (EMT) and is related to an increased risk of cancer metastasis. At the same time, the expression of E-calmodulin was also shown to be associated with the overall survival (OS) of patients with bladder cancer, suggesting a probable survival benefit, which implies that E-calmodulin expression might be a prognostic factor for life expectancy in patients with bladder cancer [78]. Inoue and colleagues investigated the expression of angiogenic- and invasion-related genes in 55 UTUC patients who had undergone RNU and found that the expression of E-cadherin is associated with bladder-specific recurrence [78]. Favaretto RL et al. evaluated 678 patients with UTUC treated with RNU and they found that reduced E-calmodulin expression is associated with a worse RFS using a univariate analysis (*p* < 0.001).

#### 3.4.2. Forkhead Box O3A

Forkhead box O3A (FOXO3A) belongs to the FOXO protein family and is located on human chromosome 6q21. It functions generally as an important transcriptional regulator involved in DNA damage repair, cell cycle regulation, apoptosis, and the cellular stress response [79]. Zhang G et al. examined the expression level of FOXO3A in 107 UTUC patients and found that the RFS was significantly shorter in patients with UTUC with low FOXO3A expression compared to that of the high-expression group (HR = 2.385, *p* = 0.004) [79]. Several studies have also demonstrated that downregulation of FOXO3A expression could promote occurrence, metastasis, and progression for UTUC patients [80]. In addition, they found lower levels of FOXO3A expression in UTUC tissues than they did in normal tissues [79]. Since FOXO3A is a recognized class of anti-cancer genes, its low expression might be related to the susceptibility of UTUC patients for recurrence and metastasis.

#### 3.4.3. HER2

HER2 is a member of the epidermal growth factor receptor (EGFR) family and is involved in cell proliferation and differentiation. Its overexpression is frequently associated with tumor growth and metastasis, therefore, HER2 has been used as an antitumor therapeutic target in some cancer patients [81]. A multicenter retrospective study by Soria F et al. including 732 patients with UTUC after RNU found that HER2 was overexpressed in 35.8% of patients. In another multivariate analysis, HER2 overexpression was also associated with IVR (*p* = 0.04) [82].

## 4. Current Treatment Measures for UTUC-BC

**Figure 2:**
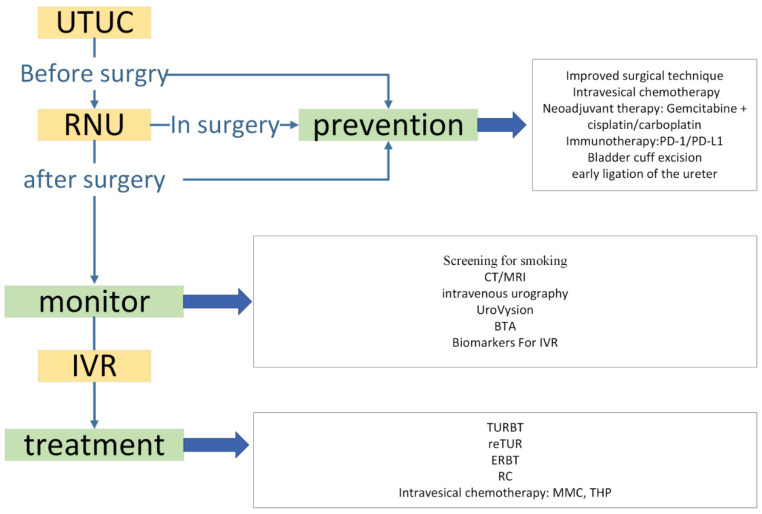
Current diagnosis and treatment strategy for patients with UTUC-BC. UTUC: upper tract urothelial carcinoma; RNU: radical nephroureterectomy; IVR: intravesical recurrence; TUR-BT: transurethral resection of bladder tumor; ReTUR: repeat TUR-BT; ERBT: en bloc resection of bladder tumor; RC: radical cystectomy; MMC: mitomycin C; THP: pirarubicin.

Currently, not a lot is known about the natural course of bladder cancer recurrence after UTUC [8]. It is not clear about the frequency and the specific time frame for which superficial bladder cancer may progress to an invasive disease; therefore, we recommend that the possible management strategy involves postoperative prevention, surveillance, and post-recurrence treatment should be taken in consideration (see Figure 2).

### 4.1. Prevention

#### 4.1.1. Surgical Techniques

Regardless of the tumor location, ORNU with bladder cuff excision has consistently been the standardized surgery in the management of patients with high-risk UTUC, and we recommend dissecting the ipsilateral medial cord ligament and lowering the ipsilateral bladder to facilitate the dissection of the whole distal segment of the ureter. The majority of the published works suggested that a minimally invasive approach might bring a more favorable perioperative outcome [84]. To lower the risk of tumor recurrence, oncological principles must be followed throughout the procedure, including the avoidance of access to the urinary tract, the avoidance of direct contact of instruments with the tumor, and using internal capsules to extract the specimens to prevent tumor seeding [85], the clamping of the ureter localized at the distal part of the UTUC tumor at an early stage to restrict the seeding of tumor cells into the bladder cavity in a downstream direction during renal and ureteral manipulation [72], and the resection of the upper urinary tract (kidney, ureter, and bladder cuff) intact [46]. Avoiding incomplete resection and ensuring negative surgical margins might contribute to lowering the recurrence rate. In addition, the surgery duration should be reasonably controlled to avoid an excessive operative time, which might cause the possibility of intracavitary metastasis and seeding tumor cells [70]. For tumors with pT > 2 stage, concomitant CIS, or extensive necrosis, surgeons should be more careful during the perioperative period [15,59]. It was mentioned previously that the involvement of lymph nodes is a risk factor for IVR [15]; therefore, we recommend that lymph node dissection should routinely performed for UTUC patients with pT > 2 stage, especially for high-risk patients. Given that the ureter is divided into upper, middle, and lower segments, the possibilities for lymph node dissection are very variable and would depend on the patient’s individual condition. Ureteroscopic biopsy and dialysis were mentioned earlier as independent risk factors for IVR [74,86], so minimizing the number of ureteroscopies and the number of dialysis preoperatively might also reduce IVR.

#### 4.1.2. Intravesical Treatment

Intravesical therapy refers to the local adjuvant therapy in which chemotherapeutic drugs are instilled into the bladder cavity through a catheter to inhibit the growth of cancer cells in the bladder. The goals of local instillation therapy in UTUC are to reduce the risk of tumor recurrence and progression and to treat CIS [87]. The commonly used drugs are: mitomycin C (MMC), gemcitabine (GEM), pirarubicin (THP), etc. It has been shown that an immediate single bladder instillation of MMC prior to RNU or partial ureterectomy (within 3 h) could reduce the risk of bladder recurrence [88]. Alternatively, intraoperative bladder instillation of MMC is feasible and is not associated with the risk of complication [89]. Fang D et al. and Hwang EC et al. performed a meta-analysis including seven randomized controlled trials, and they found that early postoperative intravesical chemotherapy with MMC and THP could reduce the risk of bladder tumor recurrence within the first year after RNU [90,91]. Ito et al. treated UTUC patients with a single bladder instillation with 30 mg THP within 48 h after RNU and showed fewer bladder recurrences in patients who received THP instillation compared to those of the patients in the control group [92]. Due to existing studies, the timing of intravesical therapy also plays a differential role in the outcome. Noennig et al. compared intraoperative and postoperative bladder instillation by MMC, and they found that the one year bladder recurrence rate was significantly lower in the intraoperative group than it was in those patients who received postoperative MMC instillation [93]. In the timing of postoperative titration, a single dose of MMC given within 24 h after RNU to prevent recurrence was demonstrated to be more effective than delayed intravesical titration is within 48 h or 2 weeks postoperatively [94]. In addition to the timing of instillation, the frequency of instillation also affects the IVR of patients with UTUC. In a study by Huang Y et al., 270 patients were divided into three groups, which included multiple, single, and no instillation groups. These patients were instilled with epirubicin (30–50 mg per instillation, 125 patients), pirarubicin (30–50 mg per instillation, 89 patients), or mitomycin C (20–40 mg per instillation, 15 patients). The patients in both instillation groups were found to have a significantly lower recurrence rate compared to that of the no instillation group (13.1 vs. 25.4% vs. 41.5%, *p* = 0.001). Multiple instillation group had a higher bladder RFS rate than the single instillation group did [95]. These findings might suggest that the use of early and multiple intravesical treatment in the perioperative period could effectively reduce the probability of IVR for patients with UTUC.

The use of adjuvant intracavitary therapy has increased in recent years, with BCG being one of the first, and perhaps, the most studied adjuvant therapies. The use of BCG for the treatment of CIS is largely considered to be the standard of care in those who meet the criteria for intermediate or high-risk non-muscle invasive bladder cancer. Its use is supported by the American Urological Association (AUA) and European Association of Urology (EAU) guidelines. However, the efficacy of upper urinary tract remains uncertain and varies by dose variation, unique delivery mechanisms, and indication [96]. The study conducted by Rastinehad et al. reported 50 patients who received BCG instillation for the treatment of UTUC at Ta/T1 stages. However, there did not have any statistical significance between UTUC patients who received and who did not receive adjuvant BCG therapy [97]. The use of BCG might be more appropriate for CIS, as Carmignani et al. have shown that the induction process of BCG could convert a positive cytology to a negative one, with a mean recurrence rate of 32% at 19–57 months of follow-up. However, cytology negativity alone was not sufficient as a sign of remission [98].

In addition to intravesical instillation, neoadjuvant chemotherapy (NAC) has become a treatment option that has been received a lot of attention in recent years. Wu Z et al. analyzed 24 studies and found that NAC had a higher survival rate and better pathological response compared to those of surgery, but there were no more significant advantages compared to those of surgery plus adjuvant chemotherapy [99]. Therefore, the specific treatment modality and timing of NAC needs to be explored by more evidence-based research. Zennami K et al. studied a total of 184 UTUC patients grouped by whether or not they received NAC before RNU and found that high-risk UTUC patients who received NAC treatment had a significantly higher 5 year RFS than the controls did (80% vs. 61%, *p* = 0.001). A higher OS was also observed in patients with disease-staged ≤cT2 who underwent the NAC treatment (*p* = 0.019) [100]. Similarly, Shigeta K et al. studied 89 patients with UTUC who received NAC or conventional adjuvant chemotherapy and found that the NAC treatment before RNU could significantly improve RFS more than treatment with chemotherapy could (*p* = 0.039) [101]. Due to the nephrotoxicity of platinum-containing drugs, preoperative NAC, such as chemotherapy with gemcitabine + carboplatin and immunotherapy with PD-1/PD-L1 immunosuppressants was encouraged to optimize the surgical outcomes [102], especially for UTUC patients with a poor renal function. So, if the patient has normal renal function, the implementation of regimens with cisplatin instead of carboplatin could bring about better therapeutic results [103]. However, if the patient has poor renal function, then platinum-containing drugs should be avoided. Additionally, immunotherapy also plays a positive role in the prognosis of UTUC patients. Fradet Y et al. analyzed 542 UTUC patients treated with pembrolizumab or conventional chemotherapy, and they found that the one year OS rates and progression-free survival rates were higher in the UTUC patients group treated with pembrolizumab (44.2% and 12.4%, respectively) than they were in the chemotherapy group (29.8% and 3.0%, respectively), with a lower incidence of associated adverse events [104].

### 4.2. Monitoring during the Follow-Up

- Screening for smoking: Smoking is one of the risk factors for recurrence, as mentioned earlier. Crivelli JJ et al. analyzed six studies, estimating the effect of smoking for patients with UTUC after receiving RNU. Most of the studies were found a statistically significant relationship between smoking and IVR. The studies also found that smoking is associated with cancer-specific mortality for patients with UTUC-BC [31], so screening for smoking is also essential.
- Imaging: Computed tomography (CT) and intravenous urography of the bladder and ureter should be performed at least once a year. If necessary, MRI should also be added into the monitoring plan.
- Endoscopy: patients with UTUC must undergo endoscopic surveillance after RNU, and the surveillance program lasts for at least 5 years, with flexible cystoscopy recommended for the surveillance of male patients [14].
- Molecular biomarkers: Various molecular biomarkers can be used to help detect recurrent bladder cancer: e.g., tumor factors, UroVysion, and BTA tests. Using Kaplan–Meier analysis, Guan B et al. showed that UTUC patients with positive UroVysion results were more likely to develop IVR during the follow-up (*p* = 0.077). These data suggest that the urinary UroVysion test may be a powerful tool for predicting the risk of IVR in patients with UTUC [105]. Walsh et al. performed a study to evaluate the effectiveness of the BTA test in patients with UTUC and found that the sensitivity of the BTA was 82% and the specificity was 89%, which were significantly better than those of the urinalysis in the same group of patients (11% and 54%, respectively) [106]. However, the study conducted by Białek Ł et al. found moderate diagnostic accuracy when they were detecting bladder cancer for patients with UTUC by BTA [107]. Therefore, more evidence is needed for BTA to detect the occurrence of IVR in patients with UTUC. Tumor factors such as E-calmodulin and FGFR3 in molecular-specific factors have been shown to correlate with IVR, so these indicators can also be evaluated during the follow-up period.

If a patient meets more of the above IVR risk factors, the frequency and length of follow-up should be increased to give appropriate consideration for the patient’s specific situation.

### 4.3. Treatment

Bladder cancer and UTUC, although they are similar, are not identical in terms of biological nature and prognosis. As only a little is known about the natural course and disease characteristics of UTUC-BC, the frequency and specific time frame for the possible progression of superficial bladder cancer to invasive disease cannot be estimated either [108]. Therefore, even though some studies have investigated risk factors for the development of IVR in patients with UTUC, there have not been large-scale studies of the treatment strategies for patients with UTUC-BC [9,15,29]. Consequently, the current management for patients with UTUC-BC is similar to the current guideline-based treatment strategies for patients with primary bladder cancer [9,108]. For NMIBC patients with a history of UTUC (UTUC-NMIBC), transurethral resection of the bladder tumor (TUR-BT) remains the initial treatment option. For MIBC patients with a history of UTUC (UTUC-MIBC), radical cystectomy (RC) is commonly recommended [9]. Since NMIBC and MIBC infiltrate different tissue layers, as shown in Figure 1, the treatment methods for them are also different, and we mainly focus on the treatment for patients with UTUC-NMIBC in this review article.

#### 4.3.1. TUR-BT

A study by Wu J et al. showed poorer outcomes among UTUC-NMIBC patients after receiving RC, with a one year overall survival (1 yr OS) of 81.8% and a three year overall survival (3 yr OS) of 56.1%, while the patients undergoing TUR-BT had relatively good outcomes (1 yr OS: 86.6%; 1 yr OS: 65.6%) [9]. TUR-BT is both the first choice management option and an important diagnostic approach for patients with UTUC-NMIBC, contributing to a prolonged RFS for the patients. Mariappan et al. found that the lack of bladder detrusor in the specimen, as well as the presence of a residual tumor, was significantly associated with an increased risk of early recurrence in the bladder [109], which made the complete excision of tumors containing bladder detrusor particularly important. Two multifactorial analyses found that tumor concomitant CIS at the time of first IVR was an independent risk factor for UTUC-BC progression [110,111]. Therefore, if a patient was found to have concomitant CIS during surgery, more attention should be paid to the complete resection of the tumor specimen and to restrictively follow oncologic principles during surgery. There was also a reduced recurrence rate when narrow band imaging (NBI) was used during TUR-BT [112].

#### 4.3.2. En Bloc Resection of Bladder Tumor (ERBT)

Tanaka N et al. investigated 241 patients with UTUC-BC after receiving RNU. Among them, the cumulative incidence rates of recurrent IVR at 1 and 5 years after treatment were 31.0% and 48.4%, respectively [111]. For the treatment of patients with such a high recurrence rate, ERBT is gradually becoming an alternative treatment to conventional TUR-BT. ERBT can obtain a complete bladder tumor specimen, allowing the pathologist to make a more accurate diagnosis of the incision margin and depth of infiltration, with it being conducive to acquiring accurate pathological staging and achieving clinical significance for postoperative bladder perfusion protocols, prognosis, and individualized follow-up program [113]. For patients with UTUC-NMIBC, ERBT was more feasible, safer, with fewer intraoperative complications than those of conventional TUR-BT, and it resulted in less remaining tumors and was unlikely to be replaced by TUR-BT [114]. It is more likely that the secondary resection could be avoided by good en block resection and might gradually become the main therapeutic modality for patients with UTUC-NMIBC in the future. Additionally, with the development of medical laser technology, there are more wide-spread lasers being used in TUR-BT, and some studies argued that TUR-BT using lasers could achieve more satisfactory treatment effects with a better prognosis than traditional electric TUR-BT can.

#### 4.3.3. Secondary Resection

A considerable number of patients with UTUC-NMIBC will experience tumor recurrence after electrotomy due to factors such as tumor stage, size, numbers, and the surgical skill of the surgeon. Therefore, for those recurrent patients, we need to repeat TUR-BT (reTUR), which requires the resection of the basal part of the original tumor area (including the surrounding mucosal inflammatory edema area) and the suspected tumor site. It is necessary to resect into the deep muscular layer of the bladder. Meanwhile, it is advised to make multiple randomized biopsies from the bladder wall. A reTUR can increase the RFS, improve the outcomes after BCG treatment, and provide prognostic information [115]. Because there are only a few surgical data about patients with UTUC-NMIBC, surgeons should decide when to perform reTUR based on the patient’s individual characteristics (e.g., concomitant CIS, etc.).

#### 4.3.4. Intravesical Chemotherapy

There are surgical options for both types of UTUC-BC, yet there are only a few data showing improved survival in UTUC-BC patients treated with these therapies [9]. Two multifactorial analyses have shown that the failure to perform intravesical therapy is an independent risk factor for disease progression for patients with UTUC-BC [12,111]. Therefore, intravesical chemotherapy is essential in the treatment program. Intravesical instillation has been shown to be effective by destroying circulating tumor cells after TUR-BT by ablating tiny residual or neglected tumor cells at the resection site [116].

It has been shown that tumors in patients with UTUC-BC respond more poorly to BCG than those in the patients with primary BC do [11]. Therefore, instillation drugs with higher sensitivity should be selected for patients with UTUC-B, and normal saline with MMC, epirubicin, or pirarubicin showed beneficial effects [117]. In a randomized controlled trial, the experimental group of normal saline combined with gemcitabine was superior to the placebo control (saline) group, with significantly lower toxicity [118]. Gemcitabine has been shown to have a response rate no less than that of the existing standard MMC and has several other advantages, including lower toxicity and costs [118].

#### 4.3.5. Photodynamic Diagnosis (PDD) and Radical Cystectomy (RC)

To further improve surgical outcomes, some studies had shown that the introduction of photosensitizers for photodynamic diagnosis (PDD) during TUR-BT could improve the complete detection of tumors and reduce residual tumors more compared to that of white light cystoscopy (WLC), but it had no significant advantage over conventional WLC in terms of diagnostic accuracy [119,120]. In addition to this, Wu J et al. found that patients with UTUC-MIBC who previously received radical cystectomy (RC) did not have significantly better survival compared to those who had tumor resection by TUR-BT [9]. The above treatment modalities are roughly the same as those for primary BC. So, physicians can make an appropriate treatment strategy when they are treating patients with UTUC-BC according to the treatment guidelines for primary BC.

## 5. Conclusions

UTUC is a rare, but highly malignant, disease, with a higher change of recurrence in the bladder and distant metastasis. In this review article, we summarized the possible mechanisms for the occurrence of IVR for patients with UTUC, including the tumor implantation theory and the correlation and characteristics of UTUC-BC and primary BC. Subsequently, we analyzed the possible factors influencing the occurrence of IVR through four aspects: the patient, tumor, treatment, and molecular specific factors. We introduced the current methods for prevention and monitoring, accordingly. In addition to this, if IVR occurs in UTUC patients, even though the current therapeutic tools are roughly the same as those used to treat primary BC, we described the advantages of these therapeutic tools and the points that need more attention when one is treating patients with UTUC-BC. Here, we recommend that urologists should develop their treatment strategies according to the risk stratification of UTUC, taking into account the specific clinical characteristics of individual patients and perform long-term, risk-adapted follow-up plans. However, due to the low incidence of UTUC, existing clinical studies are inevitably limited by their sample size, selection, and processing deviation, but there were still some inconsistent findings regarding surgical details, chemotherapeutic drug selection, and endoscopy modalities. In recent years, researchers have made continuous efforts in genomics, pathogenesis, imaging technology, and clinical practice and have achieved significant results in exploring the colonial origin and intracavitary seeding theory for UTUC-BC, improving the diagnoses and treatments for those patients. We are expecting to see there will be more available biomarkers to help urological surgeons to predict or identify possible postoperative recurrence, as well as to guide appropriate treatment options. In the future, better surgical techniques and more individualized drugs will greatly improve the survival and quality of life for patients with UTUC or recurrent BC.

## Figures and Tables

**Table 1:** Main risk factors affect recurrent BC for patients with UTUC.

Categories	Risk Factors	Reference
Patient specific factors	Damaged eGFR	Kuroda K et al. [20] Xylinas E et al. [21] Rasool M et al. [22] Chowdhury R et al. [23]
	Venerable age	Xylinas E et al. [15] Chromecki TF et al. [24] Shariat SF et al. [25]
	Gender difference	Chien TM et al. [26] Chen CH et al. [27] Xylinas E et al. [15] Ploussard G et al. [28] Seisen T et al. [29]
	Smoking	Xylinas E et al. [15] Xylinas E et al. [30] Crivelli JJ et al. [31] Ehdaie B et al. [32]
	Diabetes mellitus with poor glycemic control	Tai YS et al. [33] Gao X et al. [34] Duan W et al. [35]
	Monocyte-to-lymphocyte ratio (MLR)	Liu J et al. [36] Zhang XK et al. [37]
	Neutrophil-to-lymphocyte ratio (NLR)	Mathieu R et al. [38] De Larco JE et al. [39] Vartolomei MD et al. [40] Vartolomei MD et al. [41]
Tumor specific factors	Multifocality of upper urinary tract tumors	Milojevic B et al. [42] Chen CS et al. [43] Sheu ZL et al. [44] Chromecki TF et al. [45]
	Size of upper urinary tract tumor	Kauffman EC et al. [46] Shibing Y et al. [47] Espiritu PN et al. [48] Su X et al. [49]
	Distal ureteral position	Tanaka N et al. [16] Xylinas E et al. [15] Seisen T et al. [29] Wu Y et al. [50]
	Lymph node involvement	Arancibia MF et al. [51] Xylinas E et al. [15] Roscigno M et al. [52] Novara G et al. [53] Verhoest G et al. [54] Peyrottes A et al. [55]
	Invasive pT staging	Seisen T et al. [29] Verhoest G et al. [54] Li YR et al. [56]
	papillary structure of tumors	Remzi M et al. [57] Fritsche HM et al. [58] Ishioka J et al. [59]
	Extensive tumor necrosis	Seisen T et al. [29] Zigeuner R et al. [60] Simone G et al. [61] Zhang L et al. [62]
	Concomitant carcinoma in situ (CIS)	Wheat JC et al. [63] Roscigno M et al. [64] Otto W et al. [5]
Treatment specific factors	Incomplete excision	Kauffman EC et al. [46] Zou L et al. [65] Chung JH et al. [66] Seisen T et al. [29]
	Immature laparoscopic technique	Favaretto RL et al. [67] Piszczek R et al. [68] Seisen T et al. [29] Shigeta K et al. [69]
	Surgery time	Yanagi M et al. [70] Shigeta K et al. [71]
	Early ureteral ligation	Yamashita S et al. [72] Chen MK et al. [73]
	Ureteroscopy	Sung HH et al. [74] Li YR et al. [56] Yoo S et al. [75] Loizzo D et al. [76] Ha JS et al. [77]
Molecular specific factors	E-calmodulin	E- Inoue K et al. [78]
	FOXO3A	Zhang G et al. [79] Li J et al. [80]
	HER2	Sasaki Y et al. [81] Soria F et al. [82]

## Data Availability

Not applicable.
